# Adaptation and Evaluation of the Clinical Impairment Assessment to Assess Disordered Eating Related Distress in an Adolescent Female Ethnic Fijian Population

**DOI:** 10.1002/eat.20665

**Published:** 2010-03

**Authors:** Anne E Becker, Jennifer J Thomas, Asenaca Bainivualiku, Lauren Richards, Kesaia Navara, Andrea L Roberts, Stephen E Gilman, Ruth H Striegel-Moore, for the HEALTHY Fiji Study Group

**Affiliations:** 1 Department of Global Health and Social Medicine, Harvard Medical SchoolBoston, Massachusetts; 2 Eating Disorders Clinical and Research Program, Department of Psychiatry, Massachusetts General HospitalBoston, Massachusetts; 3 Klarman Eating Disorders Center, McLean HospitalBelmont, Massachusetts; 4 University of VictoriaVancouver, Canada; 5 Department of Psychology, Boston UniversityBoston, Massachusetts; 6 Narata VillageSigatoka, Fiji; 7 Department of Society, Human Development, and Health, Harvard School of Public HealthBoston, Massachusetts; 8 Department of Epidemiology, Harvard School of Public HealthBoston, Massachusetts; 9 Department of Psychology, Wesleyan UniversityMiddletown, Connecticut

**Keywords:** eating disorder, Clinical Impairment Assessment, distress, cross-cultural, Fiji

## Abstract

**Objective:**

Measurement of disease-related impairment and distress is central to diagnostic, therapeutic, and health policy considerations for eating disorders across diverse populations. This study evaluates psychometric properties of a translated and adapted version of the Clinical Impairment Assessment (CIA) in an ethnic Fijian population.

**Method:**

The adapted CIA was administered to ethnic Fijian adolescent schoolgirls (*N* = 215). We calculated Cronbach's α to assess the internal consistency, examined the association between indicators of eating disorder symptom severity and the CIA to assess construct and criterion validity, and compared the strength of relation between the CIA and measures of disordered eating versus with measures of generalized distress.

**Results:**

The Fijian version of the CIA is feasible to administer as an investigator-based interview. It has excellent internal consistency (α = 0.93). Both construct and criterion validity were supported by the data, and regression models indicated that the CIA predicts eating disorder severity, even when controlling for generalized distress and psychopathology.

**Discussion:**

The adapted CIA has excellent psychometric properties in this Fijian study population. Findings suggest that the CIA can be successfully adapted for use in a non-Western study population and that at least some associated distress and impairment transcends cultural differences. © 2009 by Wiley Periodicals, Inc. Int J Eat Disord, 2010

## Introduction

Eating disorders have well-established public health impact in North American and European populations as evidenced by their association with both high mortality and high suicide rates.^1^,^2^ Although eating disorders have been identified among priority illnesses for child and adolescent mental health by the World Health Organization (WHO),^3^ their contribution to burden of illness in regions outside of Europe and North America is largely unknown. Assessment of distress and impairment attributable to eating disorders is essential to setting priorities for allocation of scarce mental health resources in low and middle income countries where eating disorders may be increasingly common.^4^–^6^

A number of disease-specific measures have been developed to assess quality of life and impairment for individuals with eating disorders. Three instruments—the Clinical Impairment Assessment (CIA),^7^ the Quality of Life for Eating Disorders (QOL ED)^8^ and the Eating Disorders Quality of Life (EDQOL)^9^—are brief self-report measures that show promising psychometric properties but have not yet been evaluated in non-Western populations.^10^ Because social and occupational impairment is relative to the local social context and because both expression and perception of distress are culturally mediated,^11^,^12^ it is not clear whether utility of such measures extends across diverse populations.

The primary aim of this study was to evaluate reliability and validity of a measure of distress and impairment for eating disorders, the CIA, which we adapted and translated for use in an ethnic Fijian adolescent population.

## Method

### Study Site

The study was conducted in two adjoining provinces on the main island in the Republic of Fiji, a lower middle income country undergoing rapid social change. Ethnic Fijians comprise a small-scale indigenous society—traditionally with a subsistence agriculture economy—making up approximately half of Fiji’s population. The gradual economic transition to a wage-based economy accompanied by widespread exposure to Western-based mass media has stimulated consumption of Western food and electronic products.^13^ There is no indigenous category for an eating disorder in the local nosology despite evidence of symptomatic behaviors becoming more prevalent in the past 10 years.^14^,^15^ Although primary health care services are delivered regionally, mental health resources in Fiji are largely centralized in an inpatient facility, St. Giles Hospital, in the major urban center of Suva.

### Study Sample

Study participants (*N* = 215) were a subset of a larger study population of ethnic Fijian schoolgirls, ages 15–20 enrolled in the 12 secondary schools registered in Fiji’s Ministry of Education for this administrative area when the study was designed. In the latter study, participants responded to a battery of self-report assessments and were weighed and measured. Procedures for translating and adapting the self-report assessment battery and administering it to the larger study sample are described elsewhere.^16^

Respondents comprised all study participants present in school on the day or days of the site visit following this survey, who had screened positive for symptoms associated with an eating disorder (‘‘symptomatic respondents’’) as well as 10%–15% of respondents within each school site who did not screen positive (‘‘asymptomatic respondents’’). Asymptomatic respondents were randomly selected for participation within each site among study participants present in school at Time 2.a For this study, a positive screen was defined as the presence of one or more of the following: (A) any self-reported vomiting or laxative misuse for weight control over the past month on either the EDE-Q or the Global School-based Health Survey (GSHS); (B) eight or more instances of any inappropriate measures to control weight (e.g., excessive exercise, fasting, any purging) on the EDE-Q; (C) eight or more self-reported binge episodes on the EDE-Q; (D) a global EDE-Q score > = 4; and (E) Body Mass Index (BMI) \= 17.5 kg/m.^2^

### Procedure

The primary outcome reported here, eating disorder specific distress and impairment, was assessed by a version of the CIA, adapted as an interview schedule for this study population. The CIA is self-report measure designed to be used as a companion instrument to the EDE-Q. A total of 16 items are scored on a 4-point Likert scale (with response options, ‘‘not at all,’’ ‘‘a little,’’ ‘‘quite a bit,’’ and ‘‘a lot’’). These responses correspond to numeric values 0 to 3, which are summed to calculate a global score as long as at least 12 of the items have responses.^7^,^17^

The CIA was adapted for use in this study population as follows from a previous 22-item version obtained from its authors. Four items that referenced ‘‘work’’ were rephrased to reference school or schoolwork instead after consultation with an author of the CIA (Fairburn, Personal communication, 2007). An additional two items presented a culture-specific content problem. Item 11 that referenced ‘‘sex life’’ was omitted based on ethnographic work by the first author suggesting it could be culturally insensitive in a face-to-face interview and item 16 that referred to feeling ‘‘guilty’’ was rephrased to ask about feeling ‘‘like you had done something wrong’’ because guilt has a jural—but not affective—meaning in the local cultural idiom. The 21 remaining items and their response options were then translated into the vernacular language (i.e., a local dialect understood throughout this area) by the first author, edited by a bilingual native speaker, back-translated into English by another bilingual speaker, and then reviewed to ascertain that versions were comparable and translatable with respect to conceptual content. Study staff administered the CIA orally to the participants in their choice of either English or the vernacular language following an EDE interview. Respondents could have questions posed or rephrased in the standard Fijian language for clarification if necessary. Although we had collected data for the 21-item version as described earlier, for this analysis we discarded items not in the current version of the CIA and scored the remaining items by summing numeric responses for each of 16 total items—as long as at least 12 items were present—in order to facilitate comparison of our results with other study populations. Questionnaires with missing items were prorated through mean replacement to achieve the full range of scores. The retained items for this study analysis are the same as those in the current version of the CIA except for the minor wording changes as described earlier for 2 items.

### Self-Report Assessments

We chose several self-report assessments relating to disordered eating and body satisfaction to examine their expected correlations with distress and impairment due to eating disorder symptoms. We also selected several proxies for generalized distress (i.e., not specifically related to disordered eating) to examine their correlation with the CIA.

#### Eating Disorder Examination Self-Report (EDE-Q)

The EDE-Q, adapted for this study population, was used to assess the presence and severity of disordered eating symptoms in this study.^18^ Our adaptation of the EDE-Q demonstrated adequate internal consistency, test-retest reliability, and construct validity in this study population.^16^ Global scale scores were used as a measure of symptom severity and presence of any vomiting or laxative misuse to manage weight (including use of traditional purgatives^16^), presence of binge-eating, and presence of fasting were used as indicators of disordered eating.

#### Body Figure Scale (BFS)

The BFS directs respondents to view 9 female adult figure drawings that range from very thin to obese body size and select the figure that they perceive matches their own body shape as well as the one that matches the body shape they feel is ideal. A discrepancy score can be derived by subtracting the numeric value of the ideal shape from the self-perceived shape value. Scores close to 0 reflect little or no discrepancy; positive values reflect perception of being larger than ideal whereas negative values reflect perception of being smaller or thinner than ideal.^19^ The absolute value of discrepancy scores was used as a proxy for body dissatisfaction for this analysis.

#### Fijian Body Shape Concern and Dissatisfaction Questionnaire (FBSQ)

The FBSQ is a 6-item self-report measure assessing body dissatisfaction; five items have a Likert format with four response options and one has a forced choice yes/no response option. A previous version of this questionnaire has shown acceptable internal consistency reliability in a similar study population.^20^ Higher scores reflect greater shape and weight concerns.

#### Body Esteem Scale for Adults and Adolescents (BESAA)

The BESAA is a 23-item self-report questionnaire measuring dimensions of body esteem including feelings about general appearance and bodies.^21^ For this study the subscale relating to weight esteem was used as a measure of body satisfaction. A low score on the weight esteem subscale is consistent with body dissatisfaction.

#### Center for Epidemiologic Studies Depression Scale (CES-D)

The CES-D is a 20-item self-report questionnaire designed to measure depression in the general population.^22^ Subjects are asked to indicate how often they have felt a certain way during the past week using a 4-point Likert scale. This measure has been used successfully in adolescent and in culturally diverse populations. The CES-D score was used as a measure of psychopathology related to mood disturbance and, thus, a proxy for generalized psychopathology not specifically related to an eating disorder in this analysis.

#### Global Behavioral School-based Student Health Survey (GSHS)

The GSHS is a self-report assessment of health related behaviors in school-going adolescents developed by the WHO in collaboration with the U.S. Centers for Disease Control. The GSHS is comprised of modules relating to health risk behaviors that can be adapted to the needs assessment and local cultural context across global settings.^23^ One item from the GSHS module on nutritional behaviors, relating to vomiting or laxative use in the past month was used in combination with similar items from the EDE-Q to identify study participants for eligibility for a CIA interview. In addition, four items from the module on mental health-assessing a 12-month occurrence of loneliness, worry, a 2-week episode of sadness/hopelessness, and suicidal thoughts-were used as measures of generalized distress (that is, distress not specifically related to an eating disorder or disordered eating) in this study.

#### Weight Assessment

Weight was measured on the day of the self-report assessment on an electronic scale with a digital readout in gradations of 0.2 kg. Respondents were asked to remove shoes, sweaters or jackets, and heavy jewelry prior to being weighed in a lightweight school uniform. Measured weight was adjusted by sub-tracting 0.5 kg to yield an estimate corrected for the average contribution of the clothing. Height was measured to the nearest millimeter (mm) in bare or stocking feet with a portable stadiometer, but rounded to the nearest centimeter for the majority of observations in this analysis. BMI was calculated based on the adjusted weight and height for each respondent as kilograms/m^2^ [correction made here after initial online publication]. Respondents residing in the upper and lower 5th percentiles for age-adjusted BMI were identified by WHO adolescent anthropomorphic standard.^24^ Age-adjusted BMI cut points ranged from 16.5 to 17.5 kg/m^2^ for identifying under-weight, and 26.3 to 30.0 kg/m^2^ for identifying obesity.

#### Statistical Analyses

Characteristics of the study population were summarized by assessing frequencies and means of demographic covariates, including age, BMI, urban/rural location of school, school form (grade), EDE-Q scores, and CIA scores. Performance of the CIA in this study population was assessed in four domains: (1) feasibility; (2) internal consistency reliability (for both the Fijian and English language versions); (3) criterion validity with regard to participants’ group classification on known measures; and (4) construct validity.

We assessed feasibility by evaluating the proportion of measures that were ultimately scoreable after accounting for missing data. We then calculated a Cronbach’s a coefficient, a summary measure of the shared variation among all the CIA items, to evaluate the internal consistency reliability of the CIA total score for both English and Fijian language versions of the interview.

Next, we evaluated criterion validity by dividing the sample into asymptomatic versus symptomatic groups on nine different indices of eating disorder psychopathology, and conducting a series of t-tests comparing CIA scores across groups. We hypothesized that symptomatic participants would exhibit higher CIA scores, indicative of greater functional impairment and distress, than asymptomatic participants in the following nine comparisons:

1. Respondents with self-reported presence of vomiting or laxative abuse versus those who reported none
2. Respondents with self-reported presence of herbal purgative use versus those who reported none
3. Respondents with self-reported presence of binge eating versus those who reported none
4. Respondents with self-reported presence of fasting versus those who reported none
5. Respondents with global EDE-Q scores > = 4 versus scores \4
6. Respondents with a discrepancy score > = 2 on the Body Figure Scale versus \2
7. Respondents with a BMI at or above the 95th percentile based on WHO guidelines versus those below the 95th percentile
8. Respondents with a BMI at or below the 5th percentile based on WHO guidelines versus those above the 5th percentile
9. Respondents who screened in for an interview based on eating disorders symptoms, low weight, or high EDE-Q global scores versus those chosen randomly who did not meet screening criteria

Lastly, to assess construct validity, we calculated Pearson correlation coefficients to determine the extent to which CIA scores were correlated with measures of both eating pathology (i.e., positively associated with global EDE-Q and FBSQ, and inversely associated with BESAA) as well as proxies for generalized psychopathology and distress (i.e., CES-D and relevant GSHS items). Moreover, we tested the hypothesis that eating disorder psychopathology would be predictive of CIA scores independently from generalized measures of distress not specifically related to disordered eating. We did this by fitting three linear regression models predicting mean CIA scores. In the first model, we included global EDE-Q only; in the second model, we added participant demographic covariates (age, BMI, and language of interview); finally, in the third model we added measures of generalized psychopathology and/or distress (CES-D and GSHS items).

#### Ethical Review

The administration and evaluation of the CIA was a component of a study protocol that was approved by the Fiji National Research Ethical Review Committee (FN-RERC), the Partners Healthcare Human Subjects Committee, and the Harvard Medical School Committee on Human Studies. Parental informed consent and youth assent were obtained for all study participants.

## Results

### Sample Description

Of 183 symptomatic study participants selected for an interview, 178 were present in school on the day that interviews were conducted; of these, all completed a CIA interview. Of 37 asymptomatic respondents randomly selected and present in school, all completed a CIA interview. The mean age of the sample was 16.80 years (SD = 1.10 years), and the mean BMI was 24.28 kg/m^2^ (SD = 3.30 kg/m^2^). Urban/rural distribution, school grade, and other relevant sample characteristics for those meeting symptomatic criteria and not meeting symptomatic criteria are summarized in Table 1.

**Table 1:** Demographic characteristics of 215 adolescent Fijian schoolgirls who completed CIA interviews after screening positive or negative for eating disorder symptoms

	Asymptomatic (*n* = 37)	Symptomatic (*n* = 178)
	Mean (SD)	
Age	16.86(1.21) year	16.78(1.08) years
BMI	23.71(2.86) kg/m^2^	24.40(3.38) kg/m^2^
EDE-Q Global	1.23(0.88)	2.26(1.10)
CIA	6.11(7.32)	12.70(11.21)
	Frequency (Percent)	
Community Population Density		
Urban	19(51.4)	86(48.3)
Rural	18(48.6)	92(51.7)
School Grade		
Form 3	0(0)	16(9.0)
Form 4	10(27.0)	48(27.0)
Form 5	15(40.5)	56(31.5)
Form 6	12(32.4)	58(32.6)
Language of Interview		
English	9(24.3)	65(36.5)
Fijian	28(75.7)	113(63.5)

BMI, body mass index; EDE-Q, Eating Disorder Examination Question-naire; CIA, Clinical Impairment Assessment.

### CIA Item Completion

All 215 respondents completed the interview. Of these, 208 completed all 16 items, whereas the remaining seven completed at least 12 items, allowing for the calculation of a global score for every participant. The mean CIA score was 11.57 (SD 5 10.92), and scores ranged from 0 to 43.

### Internal Consistency Reliability

Cronbach’s a calculated for respondents to both English and Fijian language questionnaires showed adequate to excellent internal consistency reliability; they were 0.91 for the English language version group, 0.94 for the Fijian language version group, and 0.93 for the entire study sample.

### Criterion Validity

**FIGURE 1:**
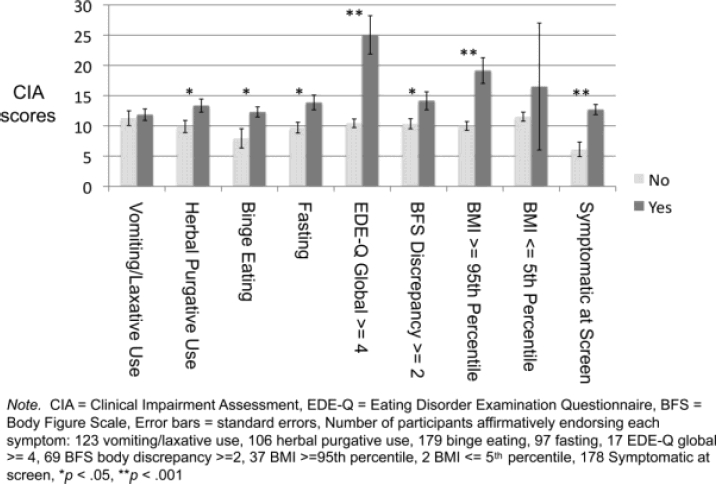
*t*-tests comparing CIA scores in symptomatic versus asymptomatic groups on nine indices of eating disorder psychopathology.

Participants classified as symptomatic on 7 of 9 indicators of eating disorder psychopathology (i.e., herbal purgative use, binge eating, fasting, elevated EDE-Q, elevated body shape discrepancy, obesity, and symptomatic screening status) scored significantly higher on the CIA than participants not classified as symptomatic (Fig. 1). The only symptomatic groups that did not exhibit elevated CIA scores in comparison to their respective asymptomatic groups were participants who self-reported vomiting/laxative use versus those who did not, and participants whose BMI fell at or below the 5th percentile for height and age versus those whose did not.

### Construct Validity

CIA scores were positively correlated with measures of eating disorder pathology as expected. Moreover, CIA scores were also correlated with measures of distress unrelated to disordered eating, as expected, with the exception of suicidal ideation, which exhibited only a trendlevel correlation with CIA, r 5 .13, p 5 .06. The observed correlations are summarized in Table 2.

**Table 2:** Correlations between CIA and continuous measures of eating disorder psychopathology and measures of generalized psychopathology and distress evaluating construct validity in an ethnic Fijian sample

Construct	r
Eating Disorder Psychopathology	
Global EDE-Q	.46**
Discrepancy Between Ideal and Actual Shape on BFS	.21*
FBSQ	.38**
BESAA-Weight Subscale	2.34**
Generalized Distress and Psychopathology	
CES-D	.32**
GSHS Loneliness	.26**
GSHS Sleeplessness due to Worry	.24**
GSHS Sadness/Hopelessness	.19*
GSHS Suicidal Ideation	.13

CIA, Clinical Impairment Assessment; EDE-Q, Eating Disorder Examina-tion Questionnaire; BFS, Body Figure Scale; FBSQ, Fijian Body Shape Con-cern and Dissatisfaction Questionnaire; BESAA, Body Esteem Scale for Adults and Adolescents; CES-D, Center for Epidemiologic Studies Depres-sion Scale; GSHS 5 Global School-Based Student Health Survey.

* *p*<.05,

** *p*<.001

Linear regression models of the relation between EDE-Q scores and the CIA are shown in Table 3. EDE-Q significantly predicted CIA scores in all three models, even when demographic covariates and measures of distress unrelated to eating pathology were added in Models 2 and 3, respectively. The standardized regression coefficient for EDE-Q in Model 3 (b 5 0.29) indicated that, independent of demographic characteristics and measures of distress and psychopathology unrelated to disordered eating, a one standard deviation increase in EDE-Q scores (reflecting eating disorder symptoms) was predictive of approximately a 1/3 standard deviation increase in CIA scores (reflecting eating disorder-specific impairment). Furthermore, in Model 3 the standardized regression coefficient for EDE-Q (b 5 0.29) was approximately 60% larger than the standardized regression coefficient for CES-D (b 5 0.18), indicating that eating disorder severity was a stronger predictor of CIA scores than was psychopathology unrelated to an eating disorder. Moreover, generalized distress (as measured by the GSHS items) was not a predictor of high CIA scores in this fully adjusted model.

**Table 3:** Linear regressions evaluating whether eating pathology predicts CIA scores when controlling for demographic covariates and generalized psychopathology and distress

	Standardized β		
	Model 1	Model 2	Model 3
EDE-Q	0.45**	0.40**	0.29**
Demographic Covariates			
Age		20.02	20.06
BMI		0.08	0.12
Language of Interview		0.07	0.03
Generalized Psychopathology and Distress			
CES-D			0.18*
GSHS Loneliness			0.10
GSHS Sleeplessness			0.02
due to Worry			
GSHS Sadness/Hopelessness			0.05
GSHS Suicidal Ideation			0.02
Model R2	0.20	0.21	0.27
Model F	F(1, 211)	F(4, 207)	F(9, 202)
R2 Change from	5 52.30**	5 13.64**	5 8.27**
		0.009	0.061
Previous Model			
F Change from		F(3, 207)	F(5, 202)
Previous Model		5 0.50	5 3.35*

BMI, body mass index; CES-D, Center for Epidemiologic Studies Depression Scale; GSHS, Global School-Based Health Student Survey; EDE-Q, Eat-ing Disorder Examination Questionnaire.

* *p*<.05,

** *p*<.001

## Discussion

Assessment of distress and impairment specific to eating disorders is critical to identifying appropriate clinical services and therapeutic strategies for individuals in need of treatment. Moreover, clarification of disease-specific burden of illness can inform health care policy decisions so that scarce mental health resource allocation most effectively meets local needs. Although eating disorders may be increasingly prevalent in populations under-going economic transition and have been designated as priority disorders by the WHO for child and adolescent mental health, little is known about the burden they impose in populations in developing areas of the world. Thus, measurement of distress and impairment specific to eating disorder symptoms can make an important contribution to the knowledge base for local mental health needs.

Our results demonstrate that the CIA is feasible, internally consistent, and informative for this ethnic Fijian adolescent female study population. Notably, the CIA measured distress related to a measure of eating disorder symptom severity (the EDE-Q global score) even when adjusted for generalized distress. The CIA also successfully discriminated between symptomatic and asymptomatic respondents for almost all of the categories examined. Reliability was comparable for the CIA in English and Fijian versions. This evidence supports the validity of the CIA as a measure of the distress and impairment associated with eating disorder pathology in this study population, even when adjusted for generalized distress and psychopathology. An illness-specific measure of distress and impairment can be a useful tool for health care providers in Fiji in assessing need for intervention in a setting in which mental health services are scarce and difficult to access. We believe that, in some cases, CIA data could also be potentially informative to educators in identifying and addressing antecedents to poor school performance. The demonstrated feasibility, reliability, and validity of the measure in a small-scale indigenous population without indigenous categories for eating disorders suggests that the CIA may also be useful across diverse populations, although further investigation is required before generalization can be made.

Measurement of illness-related distress in this particular cultural context also raises concerns about whether distress and impairment for mental illness relate to a universal standard or rather can best be understood relative to their local cultural context. For example, several findings in our study were unexpected. Specifically, low weight and vomiting and laxative use were not significantly related to CIA scores (although symptomatic participants did endorse higher CIA scores than asymptomatic participants in both instances) whereas high weight and traditional herbal purgative use were. One possible explanation is that the CIA failed to identify distress related to low weight, vomiting, or laxative misuse. Another explanationone that we find more plausible, is that social norms and cultural experience moderate distress and thus attenuate the relation between these symptoms and expressed distress. For example, social advantages of low weight may offset distress in this social environment. Individuals may perceive that thinness enhances social opportunities or may elicit relatively greater social support.^13^,^14^ Alternatively, purging may represent a somatic idiom of distress that replaces verbal communication in this population in which direct expression of complaint is discouraged for girls and women.^25^ If this is the case, then socially disenfranchised respondents may not recognize or report the relation of symptoms to distress. Future research that examines the impact of social environment on distress will be necessary to address whether and how expression and experience of distress is moderated by social norms.

There are several limitations of our study. First, the CIA was administered here as an investigator-based interview rather than as a self-report. In some cases, it was desirable to offer phrasing in a related (standard) Fijian language. As such, phrasing may have varied across raters and interviews and may have been subject to bias if the respondent had difficulty with the local vernacular language. Although we believe these elements of our protocol would have enhanced the way respondents understood the questions, it is possible that social desirability biased results in an unknown direction (i.e., either to minimize or amplify actual experience of distress). We did not assess either interrater or retest reliability of the CIA in this study population. Restricting our interviews to respondents present on the day of the site visit was a necessary compromise to logistical difficulties of travel in Fiji and could have also biased results. Also, only a small number of participants in our sample (n 5 2) met WHO criteria for under-weight, so the statistical power for detecting potential elevations in distress and impairment among low-weight participants was quite limited. Finally, our conclusions about construct validity rest on the assumption that the measures of related constructs were valid in this study population. For example, the items drawn from the GSHS as proxies for distress unrelated to disordered eating demonstrated acceptable but only modest reliability (kappas ranging from 0.41 to 0.69) (Becker et al., Reliability of the Global School-based Student Health Survey in a school-going ethnic Fijian adolescent female study population in Fiji, Unpublished). However, our data do support the reliability and validity of the EDE-Q in this study population.^16^ Moreover, we believe that the careful methods of translation and backtranslation for all the assessments used in this study, as well as data supporting their reliability (available upon request from the corresponding author) support their validity in this study population. We further note that the majority of the expected correlations between constructs were confirmed and believe that the convergence of these multiple findings strengthens our conclusion about validity of the CIA.

In sum, our study supports that the CIA is a valid measure of distress and impairment related to eating disorder symptoms in this study population. We believe that our findings suggest that some important core features of distress and impairment can be measured across diverse social environments. Our findings also underscore that sensitivity to local cultural meanings may be required to fully inform interpretation of measures of distress and impairment applied across diverse cultural settings.
